# Cytotoxicity of Aporphine, Protoberberine, and Protopine Alkaloids from *Dicranostigma leptopodum* (Maxim.) Fedde

**DOI:** 10.1155/2014/580483

**Published:** 2014-05-21

**Authors:** Ruiqi Sun, Haiyan Jiang, Wenjuan Zhang, Kai Yang, Chengfang Wang, Li Fan, Qing He, Jiangbin Feng, Shushan Du, Zhiwei Deng, Zhufeng Geng

**Affiliations:** ^1^Analytic and Testing Center, Beijing Normal University, No. 19 Xinjiekouwai Street, Haidian District, Beijing 100875, China; ^2^College of Pharmacy, Liaoning University of Traditional Chinese Medicine, Dalian, Liaoning 116600, China; ^3^State Key Laboratory of Earth Surface Processes and Resource Ecology, Beijing Normal University, Beijing 100875, China; ^4^Key Laboratory of Radiological Protection and Nuclear Emergency, Chinese Center for Disease Control and Prevention, Haidian District, Beijing 100875, China

## Abstract

Nine alkaloids with three different structural skeletons were isolated from *Dicranostigma leptopodum* (Maxim.) Fedde (Papaveraceae) by repeated silica gel column chromatography. Their chemical structures were identified on the basic of physicochemical and spectroscopic data. Among them, 10-*O*-methylhernovine (**1**), nantenine (**2**), corytuberine (**3**), lagesianine A (**4**), and dihydrocryptopine (**9**) were first isolated from this plant. With a series of cytotoxic tests, compounds **2**, **3**, and **7** displayed cytotoxicity against SMMC-7721 with IC_50_ values of 70.08 ± 4.63, 73.22 ± 2.35, and 27.77 ± 2.29 **μ**M, respectively.

## 1. Introduction

It was reported that nearly three-fifths of currently used anticancer agents were obtained from natural sources [1–3]. Therefore, utilization of ethnopharmacology is an important channel of discovering new biologically active compounds.* Dicranostigma* is a genus in the poppy family Papaveraceae, which is widely distributed in highland areas, especially in Western China. The plants of* Dicranostigma* have been used in folk medicine for treatment of tonsillitis, hepatitis, and inflammation in China for a long time [4–7]. With development of natural product chemistry, recent researches showed that* D. leptopodum* had more excellent biological activities. Extracts of* D. leptopodum* have been reported to exhibit antimicrobial activity [7], antiviral [8], antitumor [9], and anti-liver fibrosis activity [10], and anti-inflammatory activity [11]. Several compounds including alkaloids and terpenes have been reported from* D. leptopodum* [5–7]. But further investigation is necessary to find the chemical basis of activities in this plant. This work aimed to identify the active compounds by assessing the cytotoxic activity of alkaloids isolated from whole plant of* Dicranostigma leptopodum* (Maxim.) Fedde on selected cell lines. And the structural evidence related to cytotoxicity is also discussed.

## 2. Results and Discussion

**Figure 1:**
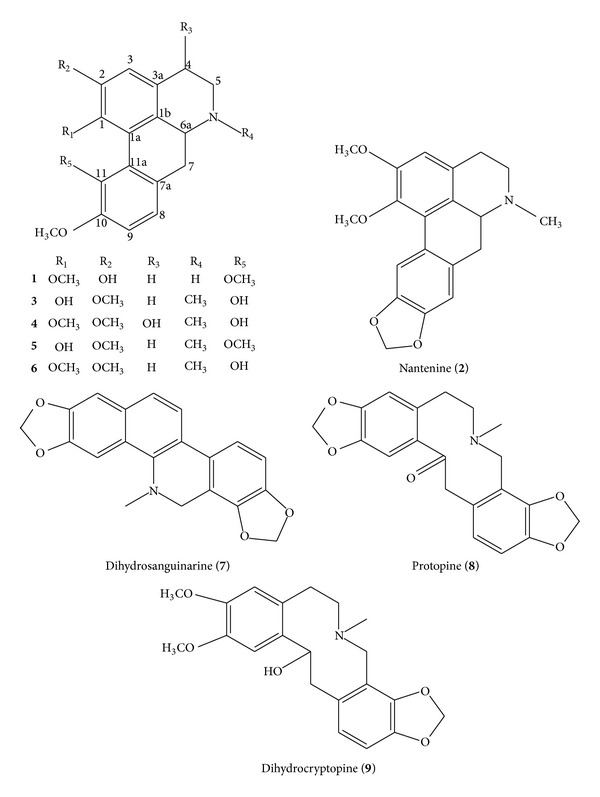
The structures of compounds** 1**–**9**.

Six aporphine alkaloids along with one protoberberine alkaloid and two protopine alkaloids were isolated and characterized from the crude extract. They were 10-*O*-methylhernovine (**1**), nantenine (**2**), corytuberine (**3**), lagesianine A (**4**), corydine (**5**), isocorydine (**6**), dihydrosanguinarine (**7**), protopine (**8**), and dihydrocryptopine (**9**). The structures of compounds** 1**–**9** were showed in Figure 1. Among them, compounds** 1**–**4** and** 9** were isolated from this plant for the first time.

In order to obtain their potential pharmacological activities, compounds** 1**–**9** were evaluated for their cytotoxicities against H1299, MCF-7, and SMMC-7721 tumor cell lines by the CCK-8 assay. The results were listed in Table 1.

Compound** 2**,** 3**, and** 7** showed their cytotoxicity against SMCC-7721. It was reported that promising effects of extracts of* D. leptopodum* were determined both in vitro and in vivo on antiproliferating of SMCC-7721 cells [12]. Therefore, the cytotoxicity showed in the report [12] might mainly relate with these constituents.

Other compounds (**1**,** 4**–**6**,** 8**, and** 9**) did not show cytotoxicity against the tested cell lines. However, there were some other biological activities of these compounds reported in the literature. This was the first time that cytotoxicity of compound** 1** was reported. Compound** 4** has been tested in vitro against human poliovirus and was found to be active with selectivity indices >14 [13]. Compounds** 5** and** 6** were reported to have weak cytotoxicity, such as nontoxic to KB cells, but showed activities in inhibiting cell proliferation of hepatocellular carcinoma [14–18]. The result was consistent with the literature that compound** 7** often showed better cytotoxicity than compound** 8** against cancer cell lines such as A549, HT-29, KB, and P-388 [19–21].

In aporphine type alkaloids, compounds** 2** and** 3** showed cytotoxicities to selected cell lines. It has been reported that compound** 2** has cytotoxicity against human colon cancer (HCT-116, Caco-2) and normal colon (CCD-18Co) cell lines [22]. And compound** 3** was reported to show strong activities in inhibiting the T or B cell proliferation and exhibited strong analgesic effects [23].

Both compounds** 3** and** 6** have 10-CH_3_ and 11-OH. It has been reported that aporphine alkaloids with a 10-CH_3_ substitution was negative to its activity related to D2 receptor, despite the presence of the critical 11-OH [24]. In addition, it was found that cytotoxicity of compound** 3** with 1-OH was stronger than compound** 6** with 1-OCH_3_. It could be inferred that cytotoxicity of aporphine alkaloids with 1-OH was stronger than those with 1-OCH_3_. And the potential drug targets of these compounds in cell might related with D2 receptor [25].

From the view of structure-activity relationship [26], it could be inferred that 1-OH together with 11-OH is necessary to exhibit cytotoxicity among aporphine alkaloids. For example, compound** 3**, having these two hydroxyl substitutions, showed certain cytotoxicity. Indeed, other aporphine compounds above showed weaker cytotoxicity against selected cell lines in this work.

## 3. Conclusions

Among compounds** 1**–**9**, it was obviously that protoberberine-type alkaloids had stronger cytotoxicity than protopine and aporphine-type ones. From the perspective of structure-activity relationship, it was expected that both 1-OH and 11-OH groups in aporphine alkaloids might be important to exhibit cytotoxic against selected cell lines while 1-OCH_3_ exhibits a negative effect to the cytotoxic. The D2 receptor in cell might be the potential drug targets of these compounds. Moreover, further study is needed to investigate the internal mechanism of alkaloids obtained from* D. leptopodum*. This may become potential basis for new antitumor drugs.

## 4. Material and Methods

### 4.1. General

^1^H and ^13^C-NMR spectra were recorded on Bruker Avance DRX 500 NMR spectrometer using CDCl_3_ (D: 99.8%, CLV, Germany) as the solvent with TMS as the internal standard. ESI-MS spectra were obtained from Bruker Q-TOF mass spectrometer. Silica gel (160–200 mesh, 200–300 mesh, Branch of Qingdao Haiyang Chemical Co., Ltd, Qingdao, China) used for column chromatography and Sephadex LH-20 was supplied by Amersham Pharmacia Biotech (Beijing, China). Analytical grade solvents were produced by Beijing Chemical Works (Beijing, China).

### 4.2. Plant Material

The whole plant (3.0 kg) of* D. leptopodum* was collected from Pingliang, Gansu Province, China (35.30°N, 107.03°E), September 2011, and identified by Dr. Liu Q.R., College of Life Sciences, Beijing Normal University. Voucher specimens (BNU-HSL-Dushushan-2011-9-25) were deposited at the herbarium (BNU) in the College of Resources Sciences, Beijing Normal University.

### 4.3. Extraction and Isolation

The dried powder (3.0 kg) was extracted by using ultrasound for three times (each for 30 minutes) with chloroform methanol (CHCl_3_/CH_3_OH) (6 L). The crude extract (160.0 g) was obtained by solvent evaporation under reduced pressure. Then silica gel column chromatography (160–200 mesh, 10.0 × 33 cm, 1000 g) was used on fractionation. Chloroform methanol solvent system (v/v ratio of chloroform, 50 : 1, 30 : 1, 10 : 1, and methanol) was used to obtain 50 fractions. Fr.42 (0.70 g) was purified by silica gel column from CHCl_3_/CH_3_OH (8 : 1) to give crystalline compound** 6** (32 mg) and compound** 5** (13 mg). Silica gel column chromatography (160–200 mesh, 2.0 × 35 cm, 100 g) of Fr.43 (1.26 g) eluting CHCl_3_/CH_3_OH (30 : 1) gave thirty subfractions (43.1–43.30). Fr.43-5 was subjected to silica gel column (160–200 mesh, 1.5 × 30 cm, 48 g) eluted with CHCl_3_/CH_3_OH (8 : 1) to afford compound** 4** (5.5 mg) and compound** 1** (4.5 mg). Fr.43-12 was subjected to silica gel column (160–200 mesh, 1.5 × 30 cm, 48 g) eluted with CHCl_3_/CH_3_OH (20 : 1) to afford compound** 7** (5 mg) and CHCl_3_/CH_3_OH (10 : 1) to obtain compound** 8** (8.3 mg). Fr.43-14 was separated by silica gel column (160–200 mesh, 1.5 × 30 cm, 48 g) eluted with CHCl_3_/CH_3_OH (10 : 1) to afford compound** 2** (5 mg). Fr.43-15 was subjected to silica gel column (160–200 mesh, 1.5 × 30 cm, 48 g) eluted with CHCl_3_/CH_3_OH (15 : 1) to obtain subfraction Fr.43-15-1 to afford compound** 9** (6.0 mg) and eluted with CHCl_3_/CH_3_OH (10 : 1) to obtain subfraction Fr.43-15-4 purified with CHCl_3_/CH_3_OH (8 : 1), yielding compound** 3** (5 mg).

*10-O-Methylhernovine (* ***1***). Light brown powder, soluble in chloroform, is with Dragendorff's test positive. ^1^H-NMR (500 MHz, CDCl_3_) *δ* ppm: 6.74 (1H, s, H-3), 3.07 (2H, d, *J* = 12.5 Hz, H-4), 2.86 (2H, d, *J* = 12.5 Hz, H-5), 3.29 (1H, m, H-6a), 2.60 (1H, m, H-7), 3.17 (1H, m, H-7), 6.83 (1H, d, *J* = 7.9 Hz, H-8), 6.86 (1H, d, *J* = 7.9 Hz, H-9), 3.92 (3H, s, 1-OCH_3_), 8.86 (1H, br, s, 2-OH), 3.74 (1H, s, 10-OCH_3_), 3.92 (1H, s, 11-OCH_3_). ^13^C-NMR (125 MHz, CDCl_3_) *δ* ppm: 142.6 (C-1), 126.0 (C-1a), 119.8 (C-1b), 152.2 (C-2), 111.3 (C-3), 130.9 (C-3a), 128.8 (C-3b), 27.4 (C-4), 41.8 (C-5), 53.6 (C-6a), 36.5 (C-7), 128.2 (C-7a), 119.4 (C-8), 111.5 (C-9), 149.8 (C-10), 144.3 (C-11), 56.0 (1-OCH_3_), 62.2 (10-OCH_3_), 56.2 (11-OCH_3_). Its NMR spectral data were in accord with the reported data [27].

*Nantenine (* ***2***). Yellow needle crystals, soluble in chloroform, exhibited a positive Dragendorff's test. ^1^H-NMR (500 MHz, CDCl_3_) *δ* ppm: 6.63 (1H, s, H-3), 4.51 (2H, s, H-7), 6.95 (1H, d, *J* = 8.0 Hz, H-8), 7.07 (1H, s, H-9), 6.92 (1H, d, *J* = 8.0 Hz, H-11), 3.88 (3H, s, 1-OCH_3_), 3.87 (3H, s, 2-OCH_3_), 2.95 (3H, s, N-CH_3_), 5.97 (2H, s, –OCH_2_–). ^13^C-NMR (125 MHz, CDCl_3_) *δ* ppm: 145.7 (C-1), 125.0 (C-1a), 121.5 (C-1b), 151.2 (C-2), 112.9 (C-3), 127.7 (C-3a), 128.2 (C-3b), 29.7 (C-4), 54.9 (C-5), 63.8 (C-6a), 38.8 (C-7), 130.9 (C-7a), 106.7 (C-8), 148.8 (C-9), 147.4 (C-10), 108.6 (C-11), 60.9 (1-OCH_3_), 55.9 (2-OCH_3_), 43.5 (N-CH_3_), 101.6 (9, 10-OCH_2_O–). The ^1^H- and ^13^C-NMR spectral data were consistent with the reported data [28].

*Corytuberine (* ***3***). Colorless columnar crystals, soluble in chloroform, were positive to Dragendorff's test. ^1^H-NMR (500 MHz, CDCl_3_) *δ* ppm: 6.71 (1H, s, H-3), 3.18 (1H, td, *J* = 14, 4 Hz, H-4), 2.69 (1H, dd, *J* = 14, 4 Hz, H-4), 3.05 (1H, m, H-5), 2.54 (1H, m, H-5), 2.99 (1H, d, *J* = 13 Hz, H-6a), 3.04 (1H, m, H-7), 2.44 (1H, d, *J* = 13 Hz, H-7), 7.01 (1H, d, *J* = 7.5 Hz, H-8), 6.92 (1H, d, *J* = 7.5 Hz, H-9), 3.92 (3H, s, 2-OCH_3_), 2.57 (3H, s, N-CH_3_), 3.76 (3H, s, 10-OCH_3_). ^13^C-NMR (125 MHz, CDCl_3_) *δ* ppm: 141.8 (C-1), 124.3 (C-1a), 130.9 (C-1b), 148.8 (C-2), 111.4 (C-3), 125.2 (C-3a), 118.9 (C-3b), 28.9 (C-4), 52.8 (C-5), 62.7 (C-6a), 35.2 (C-7), 127.9 (C-7a), 125.1 (C-8), 114.7 (C-9), 148.2 (C-10), 142.2 (C-11), 62.0 (1-OCH_3_), 44.0 (N-CH_3_), 62.5 (10-OCH_3_). The ^1^H- and ^13^C-NMR spectral data were identical with the literature data [29].

*Lagesianine A (* ***4***). Colorless columnar crystals, soluble in chloroform, gave a positive visualization to Dragendorff's test. ^1^H-NMR (500 MHz, CDCl_3_) *δ* ppm: 7.05 (1H, s, H-3), 4.59 (1H, br, s, H-4), 3.22 (1H, br, dd, *J* = 12, 1.7 Hz, H-5), 2.80 (1H, dd, *J* = 13, 3.1 Hz, H-5), 3.06 (1H, m, H-6a), 3.10 (1H, dd, *J* = 13, 3.1 Hz, H-7), 2.62 (1H, m, H-7), 6.86 (1H, d, *J* = 8 Hz, H-8), 6.89 (1H, d, *J* = 8 Hz, H-9), 3.98 (3H, s, 1-OCH_3_), 3.76 (3H, s, 2-OCH_3_), 2.67 (3H, s, N-CH_3_), 3.94 (3H, s, 10-OCH_3_), 8.76 (1H, s, 11-OH). ^13^C-NMR (125 MHz, CDCl_3_) *δ* ppm: 144.2 (C-1), 128.3 (C-1a), 119.3 (C-1b), 152.2 (C-2), 111.8 (C-3), 131.9 (C-3a), 125.8 (C-3b), 62.1 (C-4), 60.1 (C-5), 66.4 (C-6a), 35.1 (C-7), 128.8 (C-7a), 119.9 (C-8), 111.2 (C-9), 149.7 (C-10), 143.7 (C-11), 63.1 (1-OCH_3_), 55.9 (2-OCH_3_), 43.3 (N-CH_3_), 56.1 (10-OCH_3_). The above data were in accord with the literature data [30].

*Corydine (* ***5***). It is colorless columnar crystals. ^1^H-NMR (500 MHz, CDCl_3_) *δ* ppm: 6.71 (1H, s, H-3), 3.21 (1H, td, *J* = 13.4, 6.5 Hz, H-4), 2.71 (1H, m, H-4), 3.09 (1H, dd, *J* = 7, 3.5 Hz, H-5), 2.57 (1H, m, H-5), 3.06 (1H, m, H-6a), 3.08 (1H, m, H-7), 2.48 (1H, t, *J* = 13 Hz, H-7), 7.11 (1H, d, *J* = 8.3 Hz, H-8), 6.90 (1H, d, *J* = 8.3 Hz, H-9), 8.73 (1H, s, 1-OH), 3.93 (3H, s, 2-OCH_3_), 2.58 (3H, s, N-CH_3_), 3.94 (3H, s, 10-OCH_3_), 3.76 (3H, s, 11-OCH_3_). ^13^C-NMR (125 MHz, CDCl_3_) *δ* ppm: 142.4 (C-1), 123.8 (C-1a), 130.7 (C-1b), 149.3 (C-2), 111.4 (C-3), 126.4 (C-3a), 119.3 (C-3b), 28.8 (C-4), 52.7 (C-5), 62.7 (C-6a), 35.4 (C-7), 127.7 (C-7a), 124.4 (C-8), 111.0 (C-9), 151.9 (C-10), 143.9 (C-11), 56.0 (2, 10-OCH_3_), 43.7 (N-CH_3_), 62.0 (11-OCH_3_). The ^1^H- and ^13^C-NMR spectral data were consistent with the literature data [31].

*Isocorydine (* ***6***). It is colorless columnar crystals. ^1^H-NMR (500 MHz, CDCl_3_) *δ* ppm: 6.73 (1H, s, H-3), 3.20 (1H, m, H-4), 2.72 (2H, d, *J* = 17 Hz, H-4), 3.02 (1H, m, H-5), 2.49 (1H, t, *J* = 12 Hz, H-5), 2.89 (1H, d, *J* = 12 Hz, H-6a), 3.06 (1H, dd, *J* = 13.5, 3.1 Hz, H-7), 2.45 (1H, q, *J* = 13.5 Hz, H-7), 6.86 (1H, d, *J* = 8.0 Hz, H-8), 6.88 (1H, d, *J* = 8.0 Hz, H-9), 3.72 (3H, s, 1-OCH_3_), 3.94 (3H, s, 2-OCH_3_), 2.55 (3H, s, N-CH_3_), 3.92 (3H, s, 10-OCH_3_), 8.85 (1H, s, 11-OH). ^13^C-NMR (125 MHz, CDCl_3_) *δ* ppm: 142.2 (C-1), 126.0 (C-1a), 120.1 (C-1b), 151.3 (C-2), 111.1 (C-3), 129.0 (C-3a), 130.0 (C-3b), 29.4 (C-4), 52.8 (C-5), 62.9 (C-6a), 35.9 (C-7), 130.0 (C-7a), 119.0 (C-8), 111.0 (C-9), 149.5 (C-10), 144.0 (C-11), 62.1 (1-OCH_3_), 56.1 (2-OCH_3_), 43.9 (N-CH_3_), 56.0 (10-OCH_3_). The ^1^H- and ^13^C-NMR spectral data were identical with published data [32].

*Dihydrosanguinarine (* ***7***). It is pale yellow needles. ^1^H-NMR (500 MHz, CDCl_3_) *δ* ppm: 7.13 (1H, s, H-1), 7.69 (1H, s, H-4), 4.22 (2H, s, H-6), 6.87 (1H, d, *J* = 8.0 Hz, H-9), 7.32 (1H, d, *J* = 8.0 Hz, H-10), 7.71 (1H, d, *J* = 8.5 Hz, H-11), 7.50 (1H, d, *J* = 8.5 Hz, H-12), 6.08 (2H, s, 2, 3-OCH_2_O–), 6.30 (2H, s, 7, 8-OCH_2_O–), 2.64 (3H, s, N-CH_3_). ^13^C-NMR (125 MHz, CDCl_3_) *δ* ppm: 104.3 (C-1), 148.1 (C-2), 147.5 (C-3), 100.7 (C-4), 126.5 (C-4a), 142.5 (C-4b), 48.5 (C-6), 113.6 (C-6a), 144.6 (C-7), 147.1 (C-8), 107.2 (C-9), 116.2 (C-10), 127.3 (C-10a), 124.4 (C-10b), 120.4 (C-11), 123.9 (C-12), 130.8 (C-12a), 101.0 (2, 3-OCH_2_O–), 101.3 (7, 8-OCH_2_O–), 41.6 (N-CH_3_). The ^1^H- and ^13^C-NMR spectral data were in accord with published data [33].

*Protopine (* ***8***). It is colorless columnar crystals. ^1^H-NMR (500 MHz, CDCl_3_) *δ* ppm: 6.66 (1H, s, H-1), 6.93 (1H, s, H-4), 6.71 (1H, d, *J* = 7.7 Hz, H-11), 6.68 (1H, d, *J* = 7.7 Hz, H-12), 5.97 (2H, s, 2, 3-OCH_2_O–), 5.95 (2H, s, 9, 10-OCH_2_O–), 2.00 (3H, s, N-CH_3_). ^13^C-NMR (125 MHz, CDCl_3_) *δ* ppm: 110.3 (C-1), 148.0 (C-2), 146.2 (C-3), 108.0 (C-4), 135.6 (C-4a), 31.4 (C-5), 57.7 (C-6), 51.1 (C-8), 117.5 (C-8a), 146.0 (C-9), 145.9 (C-10), 106.8 (C-11), 124.9 (C-12), 128.6 (C-12a), 46.1 (C-13), 197.5 (C-14), 132.3 (C-14a), 101.2 (2, 3-OCH_2_O–), 100.9 (9, 10-OCH_2_O–), 41.6 (N-CH_3_). The ^1^H- and ^13^C-NMR spectral data were consistent with published data [34].

*Dihydrocryptopine (* ***9***). It is white powder. ESI-MS m/z: 372.5 [M + H]^+^. ^1^H-NMR (500 MHz, CDCl_3_) *δ* ppm: 7.04 (1H, s, H-1), 6.63 (1H, s, H-4), 6.63 (1H, s, H-4), 3.65 (2H, m, H-5), 3.40 (1H, m, H-6), 3.33 (1H, m, H-6), 4.31 (2H, s H-8), 6.93 (1H, d, *J* = 8.6 Hz, H-11), 6.88 (1H, d, *J* = 8.6 Hz, H-11), 3.18 (2H, m, H-13), 3.05 (1H, m, H-14), 3.86 (3H, s, 2-OCH_3_), 3.87 (3H, s, 3-OCH_3_), 5.97 (2H, s, 9, 10-OCH_2_O–), 6.30 (1H, br, s, 14-OH), 2.62 (3H, s, N-CH_3_). ^13^C-NMR (125 MHz, CDCl_3_) *δ* ppm: 107.4 (C-1), 151.3 (C-2), 146.1 (C-3), 109.0 (C-4), 130.9 (C-4a), 63.9 (C-5), 55.5 (C-6), 54.1 (C-8), 123.8 (C-8a), 147.0 (C-9), 148.5 (C-10), 125.7 (C-11), 112.3 (C-12), 124.3 (C-12a), 55.4 (C-13), 42.0 (C-14), 126.9 (C-14a), 60.8 (2-OCH_3_), 55.8 (3-OCH_3_), 101.5 (9, 10-OCH_2_O–), 42.6 (N-CH_3_). The spectrum matched the previous report [35].

### 4.4. Cytotoxicity Assay

The cytotoxicity of compounds** 1**–**9** was determined by the CCK-8 assay [36]. H1299 (nonsmall lung carcinoma), MCF-7 (breast cancer), and SMMC-7721 (liver cancer) were purchased from the Chinese Academy of Medical Sciences (Beijing, China). Doxorubicin (DOX, Adriamycin, Actavis Italy S.p.A., Beijing, China) was the positive control. All cells were grown and maintained in RPMI 1640 (Sigma, St. Louis, MO, USA) medium supplemented with 10% fetal bovine serum (Grand Island, NY, USA), 100 IU/mL penicillin (Flow Lab, Beijing, China), and 100 *μ*g/mL streptomycin (Flow Lab, Beijing, China) at 37°C, 5% CO_2_, and 90% humidity. Cancer cells were seeded in the growth medium (100 *μ*L) into 96-well microtiter plate (5 × 10^3^ cells per each well). After 4–6 h preincubation in the incubator (Forma Series *ΙΙ* Water Jacket) to allow cellular attachment, various concentrations of test solution were added and cells were incubated for 36 h. At the end of the incubation, CCK-8 reagent (Cell Counting Kit-8, Dojindo, Kumamoto, Japan, 10 *μ*L) was added into each well followed by further incubation for 2 h. The optical density (OD) was measured at 450 nm using a multiscan microplate reader (Thermo, Shanghai, China) [37]. Each determination represented the average mean of six replicates. The half-maximal growth inhibitory concentration (IC_50_) value was calculated by the line equation of the dose-dependent curve of each compound. The equation to calculate the inhibition rate was

(1) RInhibition=1−(Rdosing  cell  group−Rcontrol  group)(Rcell  control  group−Rcontrol  group)−Rsovlent.

## Figures and Tables

**Table 1:** Cytotoxicities of compounds **1**–**9** from *Dicranostigma  leptopodum* (Maxim.) Fedde.

Compound	IC_50_ (*μ*M)^a,b^		
	H1299	MCF-7	SMMC-7721
Aporphine alkaloids			
**1**	>100	>100	>100
**2**	>100	>100	70.08 ± 4.63
**3**	53.58 ± 5.47	72.30 ± 1.72	73.22 ± 2.35
**4**	>100	>100	>100
**5**	>100	>100	>100
**6**	>100	>100	>100

Protoberberine alkaloids			
**7**	28.22 ± 1.03	28.34 ± 2.00	27.77 ± 2.29

Protopine alkaloids			
**8**	>100	>100	>100
**9**	>100	>100	>100
DOX^c^	11.70 ± 1.53	7.82 ± 0.89	2.74 ± 0.34

^a^IC_50_ value was the 50% inhibition concentration and calculated from regression lines using five different concentrations in replicate experiments for six times. ^b^Solvent used in the cytotoxicity test was DMSO, and purity of compounds under the test is above 90%. ^c^Doxorubicin was used as the positive control.
